# Loss of Yeast Peroxiredoxin Tsa1p Induces Genome Instability through Activation of the DNA Damage Checkpoint and Elevation of dNTP Levels

**DOI:** 10.1371/journal.pgen.1000697

**Published:** 2009-10-23

**Authors:** Hei-Man Vincent Tang, Kam-Leung Siu, Chi-Ming Wong, Dong-Yan Jin

**Affiliations:** Department of Biochemistry, The University of Hong Kong, Pokfulam, Hong Kong, Special Administrative Region, People's Republic of China; National Institute of Diabetes and Digestive and Kidney Diseases, United States of America

## Abstract

Peroxiredoxins are a family of antioxidant enzymes critically involved in cellular defense and signaling. Particularly, yeast peroxiredoxin Tsa1p is thought to play a role in the maintenance of genome integrity, but the underlying mechanism is not understood. In this study, we took a genetic approach to investigate the cause of genome instability in *tsa1*Δ cells. Strong genetic interactions of *TSA1* with DNA damage checkpoint components *DUN1*, *SML1*, and *CRT1* were found when mutant cells were analyzed for either sensitivity to DNA damage or rate of spontaneous base substitutions. An elevation in intracellular dNTP production was observed in *tsa1*Δ cells. This was associated with constitutive activation of the DNA damage checkpoint as indicated by phosphorylation of Rad9/Rad53p, reduced steady-state amount of Sml1p, and induction of *RNR* and *HUG1* genes. In addition, defects in the DNA damage checkpoint did not modulate intracellular level of reactive oxygen species, but suppressed the mutator phenotype of *tsa1*Δ cells. On the contrary, overexpression of *RNR1* exacerbated this phenotype by increasing dNTP levels. Taken together, our findings uncover a new role of *TSA1* in preventing the overproduction of dNTPs, which is a root cause of genome instability.

## Author Summary

Peroxiredoxins are a family of antioxidant enzymes highly conserved from yeast to human. Loss of peroxiredoxin in mice can lead to severe anemia and malignant tumors, but the underlying cause is not understood. One way to derive new knowledge of peroxiredoxins is through genetic analysis in yeast. We have shown that loss of peroxiredoxins in yeast is associated with an increase in mutation rates. Here, we demonstrate that this elevation of mutation rates in yeast cells lacking a peroxiredoxin is due to increased production of deoxyribonucleoside triphosphates (dNTPs), the building blocks of DNA. Our findings suggest a new model in which compromised antioxidant defense causes accumulation of damaged DNA and activation of the DNA damage checkpoint. For yeast cells to survive DNA damage, dNTP production is increased to facilitate DNA replication, but at the price of high mutation rates. This new model could lead to a better understanding of human diseases including cancer.

## Introduction

Peroxiredoxins belong to a family of thiol-specific peroxidases widely and abundantly expressed in most living organisms [1],[2]. Through one or more redox-sensitive cysteines, peroxiredoxins not only scavenge reactive oxygen species (ROS) including peroxides and peroxynitrite [3],[4], but also function as an ROS sensor to regulate cell signaling [5]–[11]. For many peroxiredoxins, another level of regulation can be achieved through oligomerization [1],[2],[12]. In addition to their roles in peroxide reduction, peroxiredoxins are also known to possess chaperone activity [12],[13].

Loss-of-function studies in mice implicated an essential role of peroxiredoxins in antioxidant defense and tumor suppression [14]. Particularly, peroxiredoxin 1-knockout mice not only suffered from severe anemia due to oxidative stress, but were also susceptible to several types of malignant tumors [15]. Consistent with this, genome-wide screening revealed that yeast peroxiredoxin *TSA1* was a strong suppressor of gross chromosomal rearrangements and spontaneous mutations [16],[17]. In addition, a mutator phenotype was observed in yeast cells lacking one or more peroxiredoxins. The phenotype could be rescued by yeast peroxiredoxin Tsa1p or mammalian Prx1, but not by their active-site mutants defective for peroxidase activity [18],[19]. In further support of a role of *TSA1* in the maintenance of genome stability, many genetic interaction partners of *TSA1* identified through synthetic genetic array analysis were components of DNA repair machinery or DNA checkpoints [20],[21]. For example, *TSA1* was found to interact genetically with *REV1*/*REV3* and *OGG1*, which are critically involved in translesion synthesis (TLS) and the repair of oxidative DNA damage, respectively [22],[23]. However, the exact mechanism by which Tsa1p suppresses genome instability remains to be fully understood.

Intracellular dNTP levels are one important determinant of cellular response to DNA damage [24]. For yeast cells to survive DNA damage, increased dNTP production would be allowed to facilitate replication, but with a trade-off of high spontaneous mutation rate [25]. In other words, abnormally high dNTP levels are causally associated with genome instability [24],[26].

We previously demonstrated that yeast Tsa1p is a house-keeping peroxiredoxin which sufficiently suppressed the mutator phenotype [18]. Although both an aberrantly high level of ROS and an imbalance in free radical contents, which is caused by compensational activation of other antioxidants such as Sod1p [27], could underlie the mutator phenotype of *tsa1*Δ cells, additional events subsequent to the disruption of *TSA1* might also be influential in the induction of genome instability. In this study we asked whether perturbation of dNTP pools might contribute to the mutator phenotype observed in *tsa1*Δ cells. We then investigated the cause of dNTP pool expansion. Our findings suggested that constitutive activation of the DNA damage checkpoint and consequent overproduction of dNTPs are the root cause of genome instability in *tsa1Δ* cells.

## Results

### Deletion of *DUN1*, *SML1*, or *CRT1* Modulates Sensitivity of *tsa1*Δ Cells to DNA Damage

Yeast peroxiredoxin *TSA1* was found to be a strong suppressor of mutations and gross chromosomal rearrangements [16]–[18]. In addition, further deletion of another gene involved in DNA repair or DNA checkpoints caused synthetic growth defect or lethality in *tsa1*Δ cells [21],[22]. Bearing these findings in mind, here we sought to dissect the interaction of *TSA1* with the DNA damage checkpoint and particularly the machinery of dNTP synthesis, in order to understand the role of *TSA1* in the maintenance of genome stability.

**Figure 1:**
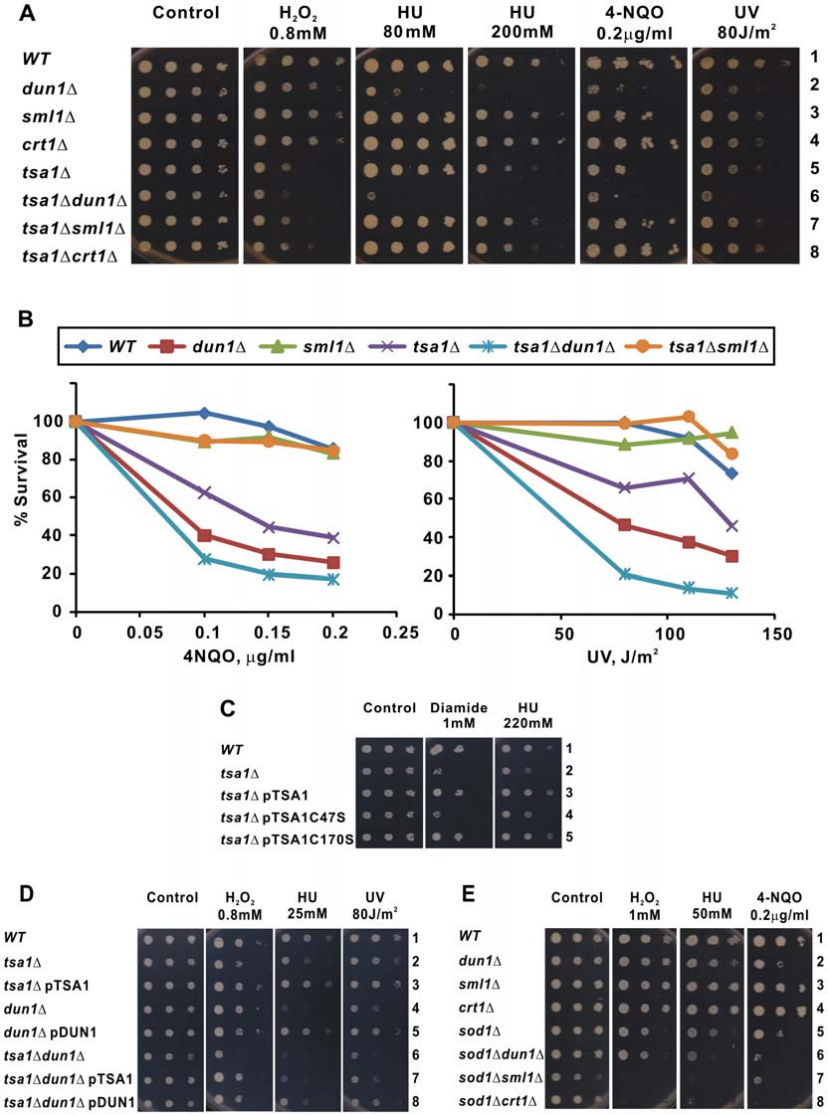
Influence of *DUN1*, *SML1*, or *CRT1* deletion on the sensitivity of *tsa1*Δ cells to DNA damage and replicative stress. (A) Spot tests. Ten-fold serial dilutions of strains BY4741 (*WT*), *dun1*Δ, *sml1*Δ, *crt1*Δ, *tsa1*Δ, *tsa1*Δ *dun1*Δ, *tsa1*Δ *sml1*Δ, and *tsa1*Δ *crt1*Δ were spotted on YPD medium containing indicated doses of H_2_O_2_, HU or 4-NQO. Cells were exposed to the indicated dose of UV after plating. Plates were incubated for 4 days at 30°C. Experiments were repeated for six times and similar results were obtained. (B) Survival curves. Logarithmically growing yeast cells, BY4741 (*WT*), *dun1*Δ, *sml1*Δ, *tsa1*Δ, *tsa1*Δ *dun1*Δ, and *tsa1*Δ *sml1*Δ, in YPD were treated with indicated doses of 4-NQO for 90 min before plating on YPD agar. For UV treatment, cells were first plated onto YPD agar followed by UV irradiation at the indicated doses. Plates were incubated for 3 days at 30°C, and then counted for survival. The number of colonies from untreated plates was taken as 100%. Experiments were repeated for three times and similar results were obtained. (C) *TSA1* catalytic cysteine mutant cannot complement the HU sensitivity in *tsa1*Δ cells. Tenfold serial dilutions of the indicated strains transformed with pRS415, pTSA1, pTSA1C47S, or pTSA1C170S were spotted on SC-Leu plates containing 2% glucose and the indicated doses of diamide or HU. Note that cells grew more slowly on SC plates than on YPD plates as shown in (A). (D) Influence of *DUN1*, *SML1*, or *CRT1* deletion on the sensitivity of *sod1*Δ cells to DNA damage. (E) Complementation of drug sensitivity in *tsa1*Δ *dun1*Δ cells by *TSA1* or *DUN1*.

We first examined the sensitivity of *tsa1*Δ cells to various DNA damaging agents. *tsa1*Δ cells were sensitive to hydroxyurea (HU), 4-nitroquinoline 1-oxide (4NQO) and ultraviolet (UV) irradiation (Figure 1A, lanes 1 and 5; Figure 1B; Figure 1C and Figure 1D, lanes 1 and 2). Re-expression of *TSA1* in *tsa1*Δ cells suppressed the sensitivity phenotype (Figure 1C and Figure 1D, lane 3). This suppression required the catalytic cysteine (Cys47) of Tsa1p, but not the C-terminal cysteine (Cys170), pointing to the importance of the antioxidant property of Tsa1p in the protection against DNA damage (Figure 1C, lanes 3–5).

The sensitivity of *tsa1*Δ cells to DNA damage prompted us to investigate further the genetic interactions between *TSA1* and components of the DNA damage checkpoint. In light of the finding that *TSA1* genetically interacts with DNA damage checkpoint genes *DUN1* and *SML1* [22], we chose these two genes and their effector *CRT1* for further analysis. Dun1p is a checkpoint kinase that phosphorylates and regulates ribonucleotide reductase (RNR) inhibitor Sml1p [28]. Dun1p also inhibits Crt1p, a transcriptional corepressor of RNR, through phosphorylation [29],[30]. Deletion of *DUN1*, *SML1* or *CRT1* in *tsa1*Δ cells exerted a significant impact on their sensitivity to HU, 4NQO and UV irradiation. Loss of *DUN1* further sensitized *tsa1*Δ cells to H_2_O_2_, HU, 4NQO and UV (Figure 1A, lanes 1, 2, 5 and 6). In support of the specificity of effect, this sensitization was reversed upon expression of *TSA1* or *DUN1* in *tsa1*Δ *dun1*Δ cells (Figure 1D, lanes 6–8). Conversely, loss of *SML1* or *CRT1* rescued the sensitivity phenotype of *tsa1*Δ cells to 4NQO and UV (Figure 1A, lanes 5, 7 and 8). It is noteworthy that such reversion of sensitivity was not observed in cells treated with H_2_O_2_ (Figure 1A, lanes 5, 7 and 8), suggesting that the effect might be specific to DNA damaging agents and was not caused directly by ROS. These observations supported the notion that *TSA1* interacts specifically with the DNA damage checkpoint in a manner that is not mediated directly through ROS.

Although the sensitivity pattern of the different mutant strains in the spot assay was highly reproducible, a more quantitative comparison of these strains is desired. Hence, survival curves of strains in the presence of 4NQO and UV were also obtained (Figure 1B). Dose-dependent killing of the strains by 4NQO and UV was observed. At all doses tested, the degrees of sensitivity of different strains to either 4NQO or UV were in the same order as shown in the spot assay. In particular, the survival curves indicated a further enhancement of the sensitivity phenotype in *tsa1*Δ *dun1*Δ versus *tsa1*Δ cells and a suppression of sensitivity in *tsa1*Δ *sml1*Δ cells (Figure 1B). Collectively, our results demonstrated that the survival of *tsa1*Δ cells under DNA damage was decreased upon deletion of *DUN1*, but enhanced when either *SML1* or *CRT1* was genetically disrupted.

We also compared the phenotypes of *tsa1*Δ cells and cells lacking Sod1p, another key antioxidant enzyme [31]. In contrast to the genetic interactions observed in *tsa1*Δ cells, deletion of *DUN1*, *SML1* or *CRT1* in *sod1*Δ cells enhanced its sensitivity to HU and 4NQO (Figure 1E, lanes 6, 7 and 8). Thus, *TSA1* and *SOD1* interact with *DUN1*, *SML1* and *CRT1* through different mechanisms.

### Deletion of *DUN1*, *SML1*, or *CRT1* Modulates Mutator Phenotype of *tsa1*Δ Cells

**Figure 2:**
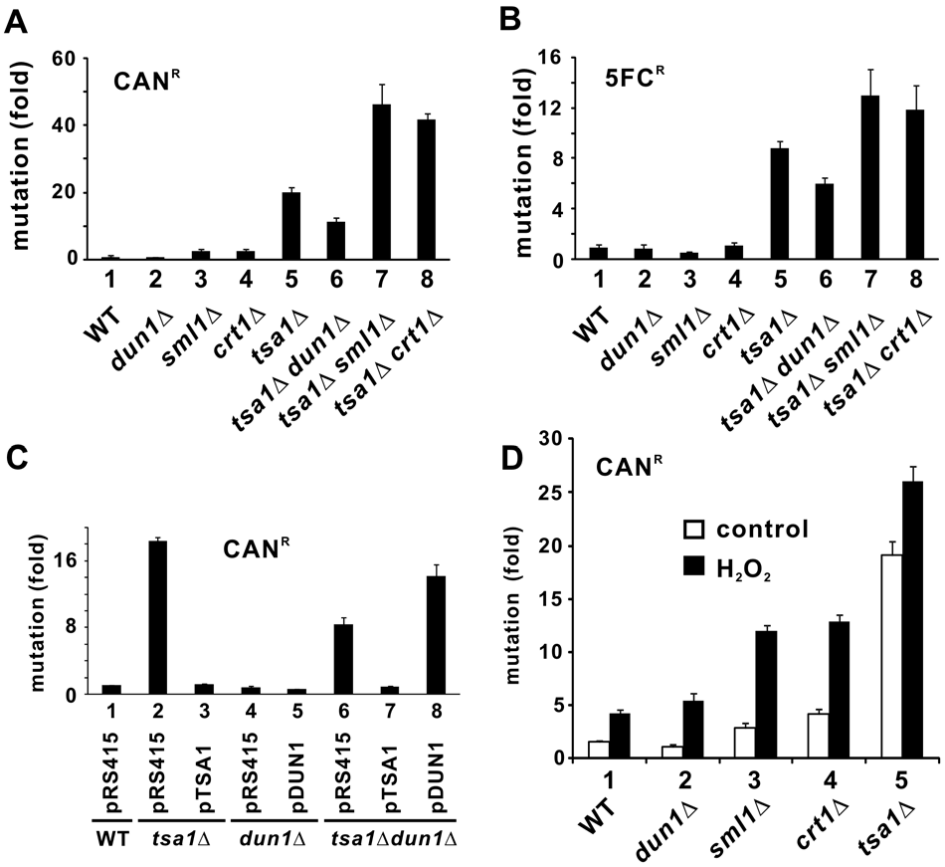
Influence of *DUN1*, *SML1*, or *CRT1* deletion on the mutation rates of *tsa1*Δ cells. The number of CAN^R^ (A) or 5FC^R^ (B) colonies on synthetic complete solid medium either lacking arginine but containing CAN (60 mg/L) or supplemented with 5FC (100 mg/L) was normalized with the total number of viable cells grown on the same solid medium without CAN or 5FC. The relative mutation rate of BY4741 cells (WT) was taken as 1.00. Results represent the average from triplicate analysis of ten independent cultures. (C) Complementation of mutator phenotype in *tsa1*Δ *dun1*Δ cells by *TSA1* or *DUN1*. The rates of spontaneous CAN^R^ mutation were calculated as in (A). (D) Influence of ROS on mutation rates. Cells of the indicated genotypes logarithmically growing in YPD were subjected to treatment of H_2_O_2_ (0.6 mM, 15 min) before plating on synthetic complete solid medium lacking arginine and supplemented with CAN (60 mg/L). The rates of spontaneous CAN^R^ mutation were calculated as in (A).

We next investigated whether compromising DNA damage checkpoint genes in *tsa1*Δ cells might also alter their mutator phenotype. In agreement with previous reports [16]–[18], *tsa1*Δ cells exhibited high rates of spontaneous mutations in both canavanine-resistant (CAN^R^) and 5FC-resistant (5FC^R^) assays (Figure 2A, Figure 2B and Figure 2D, groups 1 and 5). On the other hand, deletion of *DUN1* did not significantly affect spontaneous mutation rates (Figure 2A, Figure 2B and Figure 2D, groups 1 and 2), whereas loss of *SML1* or *CRT1* caused a mild increase in CAN^R^ mutation rates in WT cells (Figure 2A and Figure 2D, groups 1, 3 and 4). However, the disruption of *DUN1*, *SML1* and *CRT1* in *tsa1*Δ cells modulated the mutator phenotype in opposite directions (Figure 2A and Figure 2B, columns 5–8). Whereas reduction of spontaneous mutation rates was observed in *tsa1*Δ *dun1*Δ cells (Figure 2A and Figure 2B, columns 5 and 6), deletion of *SML1* or *CRT1* in *tsa1*Δ cells significantly enhanced the mutator phenotype (Figure 2A and Figure 2B, columns 1, 7 and 8). Complementation of the reduction of mutation rate in *tsa1*Δ *dun1*Δ cells by re-introduction of *DUN1* or *TSA1* further verified the specificity of effect (Figure 2C, columns 6 and 8). Thus, the mutation rates of *tsa1*Δ cells correlated with the activity of the DNA damage checkpoint.

In all individual deletion mutants, *tsa1*Δ cells displayed the highest mutation rate (Figure 2). We postulated that this might be attributed either directly or indirectly to the elevation of intracellular ROS levels in these cells [4]. If that is the case, challenging the other DNA damage checkpoint mutants with ROS might have an impact on the mutator phenotype. To test this idea, we treated the cells with low-dose H_2_O_2_ and assessed the impact on CAN^R^ mutation rates. Interestingly, mutation rates increased in WT and *dun1*Δ cells to comparable levels (Figure 2D, groups 1 and 2). In contrast, a further increase in mutability was observed when *SML1* or *CRT1* was comprised (Figure 2D, groups 1, 3 and 4). Although the mutation rates of *sml1*Δ and *crt1*Δ cells in the presence of H_2_O_2_ were still not as high as that of *tsa1*Δ cells in the absence of H_2_O_2_ (Figure 2D, groups 3–5), our results did suggest that ROS could differentially modulate the mutator phenotype of different mutants.

### Loss of *TSA1* Elevates Cellular dNTP Production

**Figure 3:**
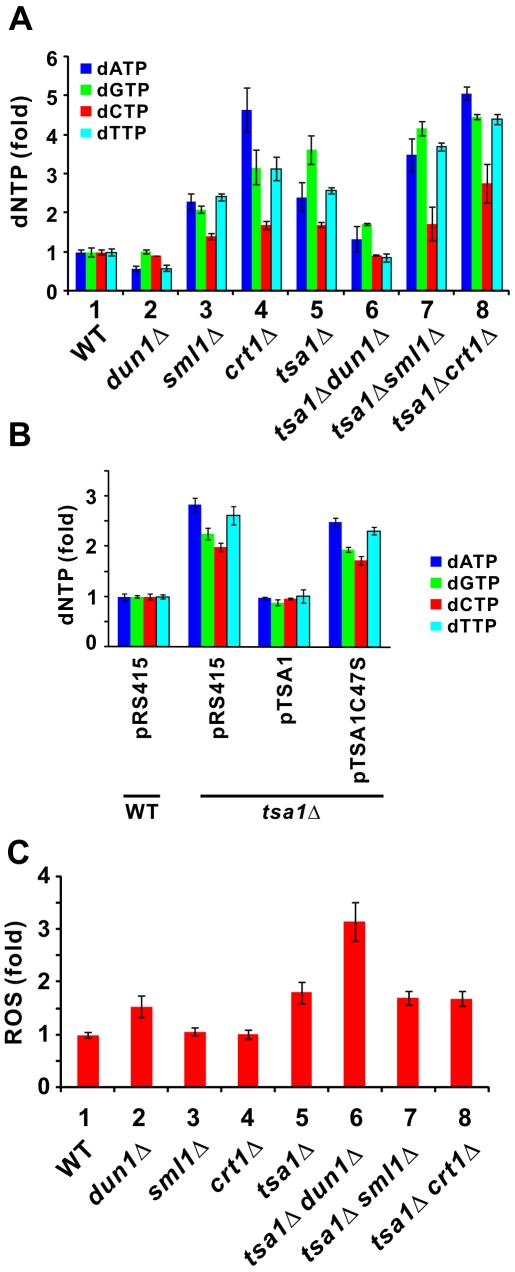
dNTP levels of *tsa1*Δ cells and influence of *DUN1*, *SML1*, and *CRT1* deletion. (A) Comparison of dNTP levels. Relative dNTP levels were determined in the indicated strains of cells growing logarithmically. (B) Suppression of dNTP pool phenotype by *TSA1*. Assays were done with WT and *tsa1*Δ strains transformed with pRS415, pTSA1, or pTSA1C47S. (C) ROS detection. Cells of the indicated strains logarithmically growing in YPD were subjected to treatment with DCF (10 µM, 45 min). Crude extracts of cells were subjected to DCF fluorescence measurement on an F-4500 spectrofluorimeter (Hitachi). The excitation and emission wavelengths were 488 and 520 nm, respectively. The reading of DCF fluorescence was normalized to protein concentration. The fluorescent intensity of BY4741 cells (WT) was taken as 1. Results represent the average from three independent experiments.

We next investigated the mechanism that underlies the correlation of DNA damage checkpoint activity in *tsa1*Δ cells with drug sensitivity and mutator phenotype (Figure 1 and Figure 2). *DUN1*, *SML1* and *CRT1* are regulators of RNR, the rate-limiting enzyme in dNTP synthesis [32]–[34]. Considered together with the model that elevated dNTP levels are required for surviving DNA damage in yeast at the price of increasing mutation rates [24], we asked whether the mutator phenotype of *tsa1*Δ cells would be due to alteration in cellular dNTP production. Thus, we measured dNTP levels in our mutants. Surprisingly, *tsa1*Δ cells produced significantly more dNTPs than wild type (WT) cells (Figure 3A, groups 1 and 5). The magnitude of dNTP overproduction in *tsa1*Δ cells was comparable to that in *sml1*Δ cells (Figure 3A, groups 3 and 5), in which the removal of Sml1p activates RNR leading to the rise in dNTP levels [28].

To shed further light on the roles of dNTP production in the generation of mutator phenotype, we compared the dNTP levels in other mutant cells. As expected, *dun1*Δ cells produced less dNTPs than WT cells (Figure 3A, groups 1 and 2), since Dun1p is required for phosphorylation and subsequent removal of the RNR inhibitor Sml1p [28]. Loss of *CRT1* was also found to increase cellular dNTP production (Figure 3A, groups 1 and 4), as Crt1p is a transcriptional corepressor of RNRs [29]. However, loss of *DUN1* reduced cellular dNTP production in *tsa1*Δ cells (Figure 3A, groups 5 and 6), whereas deletion of *SML1* or *CRT1* in *tsa1*Δ cells further increased dNTP levels (Figure 3A, groups 5, 7 and 8). Noteworthily, increased production of dNTPs in *tsa1*Δ cells could be fully complemented by Tsa1p, but not by its catalytic cysteine mutant C47S (Figure 3B). Thus, the antioxidant property of Tsa1p was likely required for preventing overproduction of dNTPs.

We then asked whether the reduction of dNTP pools in *tsa1*Δ *dun1*Δ cells would be associated with a further drop in intracellular ROS levels in the absence of *DUN1*. Interestingly, *tsa1*Δ *dun1*Δ cells exhibited a higher level of intracellular ROS over WT, *tsa1*Δ or *dun1*Δ cells (Figure 3C, columns 1, 2, 5 and 6), suggesting that the mutator phenotype in *tsa1*Δ and *tsa1*Δ *dun1*Δ cells correlates directly with dNTP production, but not generation of ROS. On the other hand, loss of *SML1* or *CRT1* did not alter the ROS levels in either WT or *tsa1*Δ cells (Figure 3C, columns 1, 3, 4, 5, 7 and 8). Thus, in addition to the accumulation of ROS, elevation of dNTP production might also contribute to genome instability in *tsa1*Δ cells.

**Figure 4:**
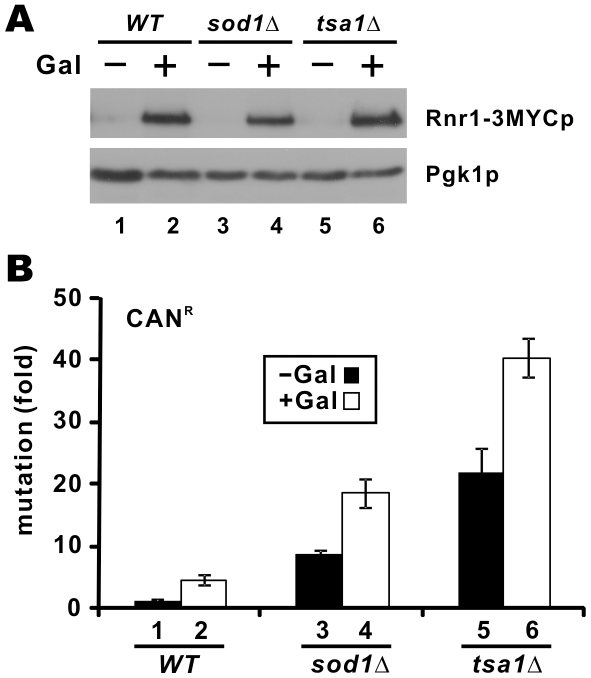
Overexpression of *RNR1* enhances mutator phenotype of *tsa1*Δ cells. BY4741 (*WT*), *sod1*Δ, and *tsa1*Δ cells carrying plasmid pGal-RNR1 were grown in SC-Ura medium supplemented with raffinose (uninduced) or galactose (induced) to mid-log phase. (A) Galactose-induced expression of Rnr1-3MYCp. Western blotting was performed with mouse anti-MYC (Roche) and mouse anti-Pgk1p (Invitrogen) antibodies. (B) Mutation rates. Experiments were carried out as in Figure 2.

While deletion of *DUN1*, *SML1* or *CRT1* has an impact on dNTP production, they are multifunctional proteins that might also affect other biological processes [28]–[30]. To address this concern, we modulated the production of dNTP more directly by overexpressing *RNR1* gene in *tsa1*Δ and *sod1*Δ cells. This overexpression has previously been shown to elevate intracellular dNTP levels substantially [24],[26]. Indeed, when we induced the expression of Rnr1-3MYCp in WT cells (Figure 4A), the spontaneous mutation rate was increased (Figure 4B, columns 1 and 2). Furthermore, overexpression of RNR1 also exacerbated the mutator phenotype in *tsa1*Δ and *sod1*Δ cells (Figure 4B, columns 3–6). This lent additional support to the importance of dNTP overproduction in the induction of genome instability.

If the mutability of *tsa1*Δ cells is indeed caused by dNTP overproduction, the mutations generated would primarily be base substitutions rather than large deletion and gross chromosomal rearrangements [24]. With this in mind, we examined the types of mutations arisen in *tsa1*Δ and other mutants (Table 1). We noted that the majority of mutations found in *tsa1*Δ cells were base substitutions (83.3%) and frameshifts (13.3%). Large deletions were very rare in *tsa1*Δ cells (3.3%) when compared with WT (13.3%) cells. In addition, all of the mutations found in *tsa1*Δ *sml1*Δ cells with high dNTP levels (Figure 3) were base substitutions (90%) and frameshifts (10%), whereas more deletions (13.3%) were detected in *tsa1*Δ *dun1*Δ cells (Table 1) with low dNTP concentrations (Figure 3). In keeping with previous findings [16], relatively more deletions (10%) were also observed in *sod1*Δ cells (Table 1). Generally, base substitutions were more prevalent in the strain when dNTP levels were high (Figure 3), whereas the incidences of deletions correlated negatively with dNTP concentrations. Therefore, the mutation spectra of *tsa1*Δ and other strains are consistent with the notion that elevation of dNTP levels is the underlying cause of genome instability in the absence of *TSA1*.

**Table 1:** Spectra and rates of CAN^R^ mutations in *tsa1*Δ and *sod1*Δ strains.

strain	type of mutation	frequency (%)	mutation rate (×10^−7^)	specific mutation rate (×10^−7^)
**wild type**	**base substitution**	**18/30 (60.0%)**	3.1	1.9
	transversion	10/30 (33.3%)		1.0
	transition	8/30 (26.7%)		0.8
	**frameshift**	**5/30 (16.7%)**		0.5
	**large deletion**	**3/30 (10.0%)**		0.3
	**complex**	**4/30 (13.3%)**		0.4
***tsa1***Δ	**base substitution**	**25/30 (83.3%)**	62.5	52.0
	transversion	15/30 (50.0%)		31.2
	transition	10/30 (33.3%)		20.8
	**frameshift**	**4/30 (13.3%)**		8.3
	**large deletion**	**1/30 (3.3%)**		2.1
***tsa1***Δ***dun1***Δ	**base substitution**	**24/30 (80.0%)**	35.5	28.4
	transversion	14/30 (46.7%)		16.6
	transition	10/30 (33.3%)		11.8
	**frameshift**	**2/30 (6.7%)**		2.4
	**3 bp deletion**	**1/30 (3.3%)**		1.2
	**large deletion**	**3/30 (10.0%)**		3.6
***tsa1***Δ***sml1***Δ	**base substitution**	**27/30 (90.0%)**	143.3	129.0
	transversion	15/30 (50.0%)		71.7
	transition	12/30 (40.0%)		57.3
	**frameshift**	**3/30 (10.0%)**		14.3
***sod1***Δ	**base substitution**	**24/30 (80.0%)**	33.9	27.1
	transversion	14/30 (46.7%)		15.8
	transition	10/30 (33.3%)		11.3
	**frameshift**	**3/30 (10.0%)**		3.4
	**large deletion**	**3/30 (10.0%)**		3.4

Mutation rates are calculated from three independent experiments, each with ten cultures. Variations are within 15% of the values.

### Loss of *TSA1* Activates DNA Damage Checkpoint Leading to Elevation in dNTP Production

Above we demonstrated the elevation of dNTP levels in *tsa1*Δ cells (Figure 3). In addition, our results also indicated the genetic interaction of *TSA1* with DNA checkpoint genes (Figure 1). This led us to further investigate whether elevated production of dNTPs in the absence of *TSA1* might be explained by the activation of the DNA damage checkpoint. As a first step, we assessed checkpoint activation by examining the steady-state levels of Rad53p, the yeast ortholog of human CHK2 kinase whose phosphorylation and activation are pivotally involved in the control of checkpoint response to DNA damage [35],[36]. Particularly, Rad53p is a master regulator of Dun1p, Sml1p and Crt1p [35].

**Figure 5:**
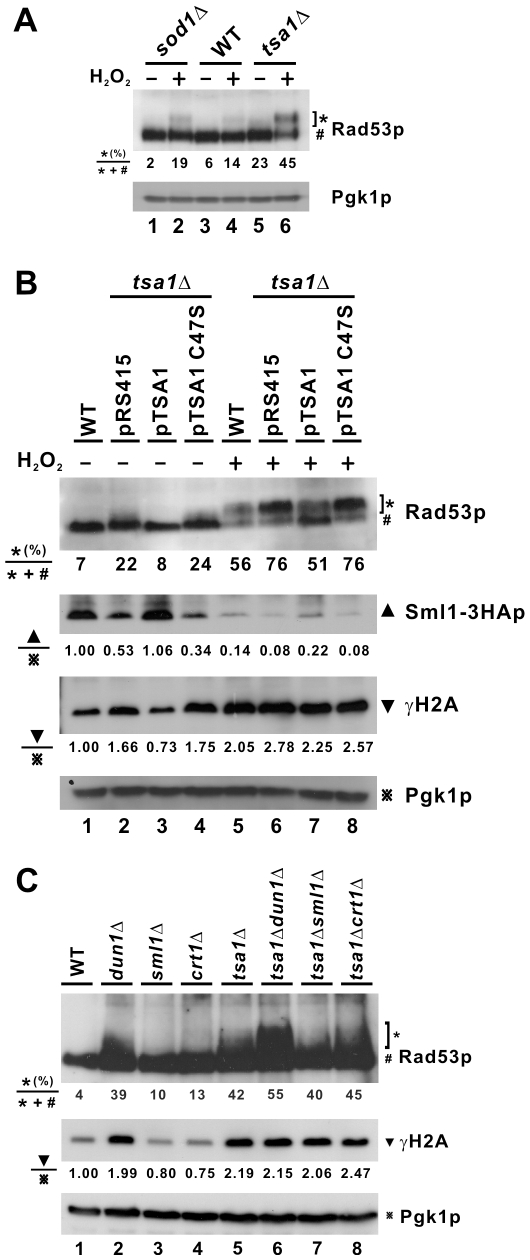
Activation of DNA damage checkpoint in *tsa1*Δ cells. (A) Western blot analysis of Rad53p. Cells of WT, *sod1*Δ, and *tsa1*Δ strains logarithmically growing in YPD were subjected to treatment with H_2_O_2_ (0.8 mM, 30 min). Western blotting was performed with goat anti-Rad53p (Santa-Cruz) and mouse anti-Pgk1p antibodies. Percentages of phosphorylated Rad53p were determined by densitometry and indicated at the bottom of the panel. (B) *TSA1* complementation assay. WT and *tsa1*Δ cells were transformed with pRS415, pTSA1, or pTSA1C47S plasmid. Western blotting was carried out with goat anti-Rad53p, mouse anti-HA (Santa-Cruz), rabbit anti-histone H2A phosphorylated at S129 (γH2A; Abcam), and mouse anti-Pgk1p antibodies. Relative amounts of Sml1-3HAp or γH2A normalized to Pgk1p were determined by densitometry and indicated at the bottom of the panels. (C) Checkpoint activation in different strains.

**Figure 6:**
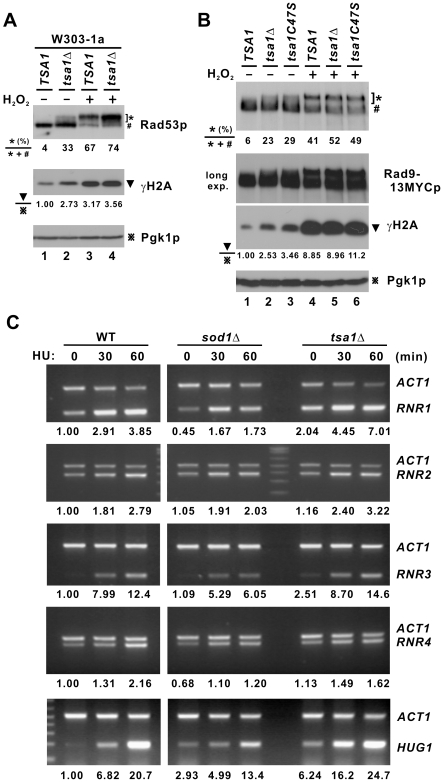
Activation of DNA damage checkpoint in *tsa1*Δ cells. (A) Western blot analysis of Rad53p. Cells of the indicated strains in W303 background logarithmically growing in YPD were subjected to treatment with H_2_O_2_ (0.8 mM, 30 min). (B) Western blot analysis of Rad9p in *tsa1*Δ and *TSA1*-complemented strains. A longer exposure (long exp.) of the Rad9p blot was also shown. (C) Semi-quantitative RT–PCR analysis of *RNR* transcripts. Logarithmically growing cells of the indicated strains in YPD were subjected to treatment with HU (200 mM) at the indicated time points. Total RNA was extracted and 3 µg of RNA was used for cDNA synthesis. PCR was performed to assess the levels of *RNR1/2/3/4*, *HUG1*, and *ACT1* transcripts. The expected sizes of the PCR product for *RNR1*, *RNR2*, *RNR3*, *RNR4*, *ACT1*, and *HUG1* are 219, 390, 199, 455, 520, and 190 bp, respectively. Relative levels of RNA determined by densitometry and normalized to the amount of *ACT1* transcript were indicated at the bottom of the panels.

**Figure 7:**
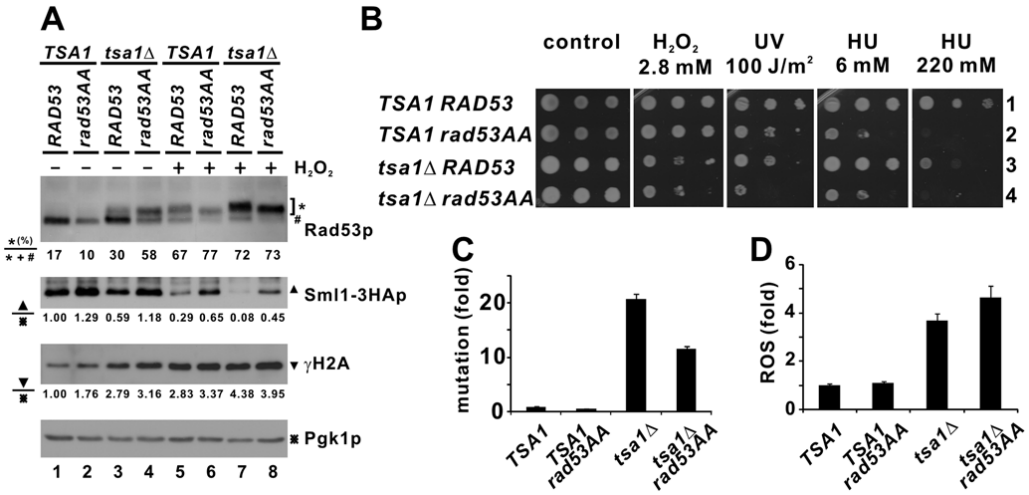
Impact of *RAD53* mutation on *tsa1*Δ cells. (A) Western blot analysis of Rad53p and Sml1-3HAp. Cells of the indicated strains in W303 background logarithmically growing in YPD were subjected to treatment with H_2_O_2_ (0.8 mM, 30 min). Western blotting was performed as in Figure 5B. (B) Spot assay. Ten-fold serial dilutions of the indicated strains were spotted on YPD medium containing the indicated doses of H_2_O_2_ or HU. Some cells were exposed to UV after plating. (C) *RAD53* mutation suppresses mutator phenotype in *tsa1*Δ cells. Mutation rates were calculated as in Figure 2B. (D) ROS detection. DCF fluorescence was measured as in Figure 3C.

In this analysis we included the *sod1*Δ control strain, in which the effectors of the Mec1p-dependent DNA damage checkpoint were previously shown to be downregulated [31]. Phosphorylated Rad53p species were more evident in *tsa1*Δ cells compared to WT and *sod1*Δ cells (Figure 5A, lanes 1, 3 and 5; Figure 5B, lanes 1 and 2; Figure 6A, lanes 1 and 2; and Figure 7A, lanes 1 and 3). This difference became more pronounced in the presence of H_2_O_2_ (Figure 5A, lanes 2, 4 and 6; Figure 5B, lanes 5 and 6; Figure 6A, lanes 3 and 4; and Figure 7A, lanes 5 and 7).

The steady-state levels of phosphorylated Rad53p in DNA damage checkpoint mutants were also compared. Whereas deletion of *DUN1* triggered phosphorylation of Rad53p (Figure 5C, lanes 1 and 2), an observable increase in phosphorylated Rad53p species was not found in *sml1*Δ or *crt1*Δ cells (Figure 5C, lanes 1, 3 and 4). Notably, loss of *DUN1* in *tsa1*Δ cells further enhanced the activation of Rad53p (Figure 5C, lanes 5 and 6), whereas *tsa1*Δ *sml1*Δ and *tsa1*Δ *crt1*Δ cells had similar levels of phosphorylated Rad53p compared to tsa1Δ cells (Figure 5C, lanes 5, 7 and 8).

In addition to Rad53p, we also checked for the status of Rad9p, a more upstream transducer in the DNA damage checkpoint pathway [37]. Rad9-13MYCp was found to be activated in *tsa1*Δ cells (Figure 6B, lanes 1, 2, 4 and 5) and this activation could not be reversed by the C47S mutant of Tsa1p (Figure 6B, lanes 2, 3, 5 and 6). As a marker for DNA double-strand breaks (DSBs) [38], the level of γH2A was also found to be elevated in *tsa1*Δ cells as compared to WT (Figure 5B, lanes 1 and 2; Figure 6A, lanes 1 and 2; and Figure 6B, lanes 1 and 2). This agrees with a recent report that *tsa1*Δ cells displayed an increased number of Rad52-YFP foci indicative of DNA damage [23]. The levels of γH2A in other DNA damage checkpoint mutant cells were also examined. Among *dun1*Δ, *sml1*Δ and *crt1*Δ cells, an elevation in γH2A level was only found in *dun1*Δ cells (Figure 5C, lane 2). In addition, disruption of *DUN1*, *SML1* or *CRT1* in *tsa1*Δ cells did not affect γH2A levels significantly (Figure 5C, lanes 5–8). Noteworthily, although phosphorylated Rad53p and γH2A were abundant in *dun1*Δ and *tsa1*Δ *dun1*Δ cells (Figure 5C, lanes 2 and 6), their mutation rates remained low (Figure 2A and Figure 2B, columns 2 and 6) plausibly due to the low levels of dNTPs (Figure 3A, groups 2 and 6). In other words, elevation of dNTP levels might be the direct cause of genome instability.

Consistent with the activation of Rad53p and Rad9p, the levels of Rad53p target Sml1-3HAp were diminished in *tsa1*Δ cells in the presence (Figure 5B, lanes 5 and 6) and absence of H_2_O_2_ (Figure 5B, lanes 1 and 2). Importantly, all of the above changes in *tsa1*Δ cells could be fully complemented by *TSA1* (Figure 5B, lanes 2 and 3), but not by its C47S mutant (Figure 5B, lanes 2 and 4). On the other hand, in agreement with previous findings [31], we did not observe a significant change of Sml1p level in *sod1*Δ cells (data not shown).

RNR is an important downstream effector of the DNA damage checkpoint which mediates the production of dNTPs [33],[34]. Since the expression of *RNR* genes is transcriptionally activated in response to DNA damage [29], we used semi-quantitative RT-PCR to determine the relative levels of *RNR1/2/3/4* transcripts in the presence and absence of HU. In this analysis we included an additional control termed *HUG1*, a target of Mec1p induced highly by DNA damage [39]. As shown in Figure 6C, *RNR* transcripts were induced to higher levels in *tsa1*Δ cells than in WT and *sod1*Δ cells. The induction of *RNR1* and *RNR3* was greatest in both untreated and HU-treated *tsa1*Δ cells. The level of *HUG1* transcript was also elevated in *tsa1*Δ cells and this was more pronounced in the presence of HU (Figure 6C). In sharp contrast, *sod1*Δ cells treated with HU showed a lower magnitude of induction of *RNR1*, *RNR3* and *HUG1* mRNAs (Figure 6C). These results obtained from *sod1*Δ cells were generally consistent with previous findings [31]. Thus, the pattern of *RNR* induction in *tsa1*Δ cells was not ascribed to a general effect caused by the lack of any antioxidant enzyme, but was highly specific. Collectively, our results suggested that loss of *TSA1* induces the activation of the DNA damage checkpoint leading to the induction of RNR and consequent overproduction of dNTPs.

### Mutation of Rad53p Suppresses Genome Instability in *tsa1*Δ Cells

If activation of the DNA damage checkpoint in *tsa1*Δ cells is really important to the generation of genome instability caused by dNTP overproduction, genetic disruption of the checkpoint would be able to reverse the mutator phenotype of *tsa1*Δ cells.

To test this hypothesis, we employed a *RAD53* mutant termed *rad53AA*, in which both T354 and T358 in the activation loop of Rad53p had been replaced by alanine, thereby abrogating the autophosphorylation activity in response to DNA damage [40]. This defective *rad53AA* allele similar to *rad53-11* is thought to be associated with reduced dNTP production due to high abundance of Sml1p [40],[41]. Thus, we set out to characterize the phenotypes of *tsa1*Δ cells carrying the *rad53AA* allele.

As documented previously [40], *TSA1 rad53AA* cells exhibited lower levels of phosphorylated Rad53p and higher abundance of Sml1-3HAp than *TSA1 RAD53* cells (Figure 7A, lanes 1, 2, 5 and 6). In response to H_2_O_2_, γH2A was induced to higher levels in all mutant cells (Figure 7A, lanes 5–8 compared to lanes 1–4). Notably, both *tsa1*Δ *RAD53* and *tsa1*Δ *rad53AA* cells showed similar basal levels of γH2A (Figure 7A, lanes 3 and 4). Although stronger Rad53p activation was observed in *tsa1*Δ *rad53AA* cells, a more pronounced Sml1-3HAp protein band was seen (Figure 7A, lane 4 compared to lane 3), suggestive of a defective DNA damage checkpoint.

We next characterized the sensitivity of these mutants towards H_2_O_2_, UV and HU. *TSA1 rad53AA* cells were sensitive to HU (Figure 7B, lanes 1 and 2) as previously described [40]; while *tsa1*Δ *RAD53* cells were sensitive to H_2_O_2_, UV and HU (Figure 7B, lanes 1 and 3) similar to *tsa1*Δ cells in BY4741 background (Figure 1, lanes 1 and 5). Resembling *tsa1*Δ *dun1*Δ cells in BY4741, *tsa1*Δ *rad53AA* cells in W303 background displayed further sensitivity to H_2_O_2_ and UV when compared to *tsa1*Δ *RAD53* cells (Figure 7B, lanes 3 and 4).

We then looked at the effect of a defective DNA damage checkpoint on genome instability in *tsa1*Δ cells. Intriguingly, *tsa1*Δ *rad53AA* cells displayed a significantly reduced (∼50%) rate of spontaneous 5FC^R^ mutations over *tsa1*Δ *RAD53* cells (Figure 7C). On the other hand, both *tsa1*Δ *rad53AA* and *tsa1*Δ *RAD53* cells had high levels of intracellular ROS over WT cells as measured by DCF fluorescence (Figure 7D). These observations suggested that *rad53AA* mutation can suppress the mutator phenotype in *tsa1*Δ cells without affecting cellular redox environment. This generally agrees with the phenotypes of *tsa1*Δ *dun1*Δ cells (Figure 1 and Figure 2), lending further support to the concept that intracellular dNTP levels are an important determinant in the induction of genome instability in *tsa1*Δ cells.

## Discussion

Here, we provided the first evidence that loss of yeast peroxiredoxin *TSA1* causes genome instability through constitutive activation of the DNA damage checkpoint leading to overproduction of intracellular dNTPs. There are two salient points in our work. First, we demonstrated the elevation of dNTP levels in *tsa1*Δ cells and its direct correlation with the mutator phenotype (Figure 2, Figure 3, Figure 4). Second, we demonstrated the activation of the DNA damage checkpoint in *tsa1*Δ cells in relation to elevated production of dNTPs (Figure 1, Figure 5, Figure 6, Figure 7). Our findings suggested a new model for the role of peroxiredoxins in the maintenance of genome integrity, which has implications in the understanding of human diseases including cancer.

In agreement with our findings on the accumulation of γH2A and activation of the DNA damage checkpoint in *tsa1*Δ cells, several lines of evidence in the literature supported the role of Tsa1p and other peroxiredoxins in the protection of cells against DNA damage. First, *tsa1*Δ cells produce significantly more ROS [4], which cause DNA and protein damage [27],[42],[43]. Second, loss of *TSA1* results in increased formation of Rad52-YFP foci, an indicator of DNA DSBs [23]. Third, *tsa1*Δ cells are highly sensitive to the functional state of DNA repair and checkpoints [22]. In particular, *tsa1*Δ is synthetically lethal with *rad51*Δ mutation, indicating that the viability of *rad51*Δ cells deficient in recombination repair requires *TSA1* function [44]. Finally, human peroxiredoxins have been implicated in cellular defense against oxidative DNA lesions [45]. In this context, the activation of the DNA damage checkpoint in *tsa1*Δ cells demonstrated in our study highlights the pivotal roles of the checkpoint in cell survival and provides an explanation for the synthetic lethality seen in various double deletion mutants involving *TSA1* and another DNA repair or checkpoint gene [21].

Deletion of *TSA1* in yeast cells has previously been shown to result in both a mutator phenotype and an increase in gross chromosomal rearrangements [16],[22],[23]. Although the causes and origin of gross chromosomal rearrangements remain poorly understood, oxygen metabolism and ROS production are implicated in the prevalence of these rearrangements in *tsa1*Δ cells [23]. Noteworthily, base substitution, but not chromosomal rearrangement, was the predominant type of mutation found in our analysis of mutation rates (Table 1). Thus, the major type of genome instability analyzed in our study is an increased rate of point mutations, but not gross chromosomal rearrangements involving more complex alterations such as translocations, large deletions and amplifications.

Our findings point to a role of dNTP levels in determining the mutation rate of *tsa1*Δ cells. Strong genetic interactions between *TSA1* and four RNR regulators *DUN1*, *SML1*, *CRT1* and *RAD53* were observed in the context of sensitivity to DNA damage (Figure 1 and Figure 7), spontaneous mutability (Figure 2 and Figure 7) and dNTP production (Figure 3). Although the catalytic cysteine of Tsa1p is required for the suppression of mutator phenotype, the mutability of *tsa1*Δ cells correlated directly with dNTP concentrations (Figure 2, Figure 3, Figure 4), but not with high ROS levels (Figure 3 and Figure 7). One plausible explanation is that the loss of *TSA1* might cause accumulation of both ROS [4] and DNA damage (Figure 5). This activates the DNA damage checkpoint through Rad53p, Rad9p and Sml1p (Figure 5, Figure 6, Figure 7) leading to transcriptional activation of RNR genes (Figure 6) and elevated production of dNTPs (Figure 3). Once at high dNTP levels, replicative and TLS polymerases by-pass DNA lesions more efficiently to promote survival, but only at the price of increasing mutation rates [24],[25]. This model implicates dNTP pool expansion as the major culprit in the induction of genome instability in *tsa1*Δ cells. Indeed, reducing dNTP levels without affecting ROS production was sufficient to reverse the mutator phenotype of *tsa1*Δ cells (Figure 3). In particular, *tsa1*Δ *dun1*Δ cells have high levels of ROS (Figure 3C), phosphorylated Rad53p (Figure 5C) and γH2A (Figure 5C). However, these cells showed a low mutation rate (Figure 2C) because the dNTP levels were also low (Figure 3A). On the contrary, increasing dNTP levels by overexpressing *RNR1* aggravated the mutator phenotype (Figure 4). Furthermore, point mutations but not deletions were predominantly found in *tsa1*Δ cells (Table 1), implicating a role for dNTP overproduction in compromising genome stability. In further support of this model, compromise of TLS polymerases also suppressed CAN^R^ mutations in *tsa1*Δ cells [23].

We found that the levels of dNTPs in *tsa1*Δ cells were as high as those in *sml1*Δ cells (Figure 3A). This finding revealed an unexpected role of *TSA1* in the maintenance of dNTP pools in eukaryotic cells. We further observed transcriptional activation of *RNR* genes in *tsa1*Δ cells (Figure 6), which could be mediated through the activation of Rad53p checkpoint. Although this might provide an explanation for the overproduction of dNTPs, exactly how Tsa1p is mechanistically involved in regulating *RNR* expression remains to be further investigated.

Consistent with previous findings [26], elevation of intracellular dNTPs over a particular threshold level by overexpressing Rnr1p can sufficiently induce a mutator phenotype (Figure 4). Plausibly, the dNTP levels in *tsa1*Δ *sml1*Δ and *tsa1*Δ *crt1*Δ cells might have reached the threshold level causing a dramatically increased mutation rate (Figure 2 and Figure 3). When the elevation of dNTP levels have not reached the threshold as in the case of *sml1*Δ, *crt1*Δ and *tsa1*Δ cells, accumulation of intracellular ROS might serve to trigger or aggravate the mutator phenotype. In *tsa1*Δ cells, ROS levels were constantly high (Figure 3C) causing severe DNA damage (Figure 5). In contrast, ROS levels were low (Figure 3C) and DNA damage was not detected (Figure 5C) in *sml1*Δ or *crt1*Δ cells. This might explain the higher mutation rate in *tsa1*Δ cells versus *sml1*Δ or *crt1*Δ cells (Figure 2). Further exacerbation of the mutator phenotype of *sml1*Δ and *crt1*Δ cells by ROS such as H_2_O_2_ (Figure 2D) lent some credence to this model.

We demonstrated the requirement of the catalytic cysteine for the ability of Tsa1p to modulate dNTP production (Figure 3). Through irreversible hyperoxidation, this residue can act as a redox sensor, which triggers the switch of peroxiredoxin from peroxidase to chaperone activity under stress [12],[13]. In this connection, it would be of great interest to understand whether and how the chaperone activity of Tsa1p might be involved in the regulation of dNTP production.

Activation of Rad53p by upstream kinase Mec1p requires adaptor proteins Rad9p and Mrc1p [46],[47]. We noted that Rad53p phosphorylation was dramatically increased in *tsa1*Δ versus WT cells (Figure 6A and Figure 7A). In contrast, the increase in Rad9p phosphorylation in the absence of *TSA1* was less pronounced (Figure 6B). Although additional experiments are required to investigate the cause of this difference between Rad53p and Rad9p, one possibility is that the deletion of *TSA1* might exert a stronger effect on Mrc1p activity.

Hypermutability or genome instability is a hallmark of cancer [48]. Mammalian *Prx1* is a candidate tumor suppressor gene [15],[49]. Because peroxiredoxins are highly evolutionarily conserved proteins, an understanding of the mechanism by which yeast Tsa1p protects cell from genome instability might derive novel insight into the tumor suppressive role of Prx1 in mammalian cells. Our work demonstrates the importance of high dNTP levels in the mutability of *tsa1*Δ cells. Further analysis of dNTP concentrations of Prx1-null mouse cells will reveal whether increased production of dNTPs might be a general mechanism for the generation of genome instability in higher eukaryotes.

## Materials and Methods

### Strains and Plasmids

*S. cerevisiae* strains BY4741 [50] and W303-1a, and their isogenic strains (Table 2) were used. All knockout mutants were constructed by one-step gene deletion method [51]. Primers were listed in Table 3. Expression vector for *DUN1* was derived from pRS415. Expression plasmids for *TSA1* and its mutants have been described [18].

**Table 2:** Yeast strains.

name	parent	genotype	reference
BY4741		*MATa his3*Δ*1 leu2*Δ*0 met15*Δ*0 ura3*Δ*0*	[49]
HMY001	BY4741	*tsa1*Δ::HIS3*	This work
3798^a^	BY4741	*dun1*Δ::*KanMX4*	[49]
512^a^	BY4741	*sml1*Δ::*KanMX4*	[49]
4125^a^	BY4741	*crt1*Δ::*KanMX4*	[49]
6913^a^	BY4741	*sod1*Δ::*KanMX4*	[49]
HMY002	3798	*dun1*Δ::*KanMX4 tsa1*Δ::HIS3*	This work
HMY003	512	*sml1*Δ::*KanMX4 tsa1*Δ::HIS3*	This work
HMY004	4125	*crt1*Δ::*KanMX4 tsa1*Δ::HIS3*	This work
HMY005	3798	*dun1*Δ::*KanMX4 sod1*Δ::HIS3*	This work
HMY006	512	*sml1*Δ::*KanMX4 sod1*Δ::HIS3*	This work
HMY007	4125	*crt1*Δ::*KanMX4 sod1*Δ::HIS3*	This work
HMY1001	HMY001	*tsa1*Δ::HIS3* RAD9-13MYC::*KanMX4*	This work
W303-1a		*MATa ade2 ura3 trp1 leu2 his3 can1*	
cy7075	W303-1a	*SML1-3HA*::*HIS*	[40]
cy7096	cy7075	*SML1-3HA*::*HIS rad53AA*::*KANMX4*	[40]
HMY008	cy7075	*SML1-3HA*::*HIS tsa1*Δ::*TRP1*	This work
HMY009	cy7096	*SML1-3HA*::*HIS rad53AA*::*KANMX4 tsa1*Δ::*TRP1*	This work

a Purchased from Research Genetics [49].

HIS3* designates the *HIS3* allele from *Saccharomyces kluyveri*.

cy7075 and cy7096 were gifts from Dr. Achille Pellicioli (FIRC Institute of Molecular Oncology Foundation, Milano, Italy).

**Table 3:** Oligonucleotide sequences for RT–PCR and site-directed mutagenesis.

name	sequence	expected size (bp)
RNR1-RT-F	ATTTCGTGCCCGCAGC	219
RNR1-RT-R	TTCCTCATCA TCAACGATGGG	
RNR2-RT-F	ATGCCTAAAGAGACCCCTTCCA	390
RNR2-RT-R	CTCGTTTTCGTTCATTCTGTTGTTC	
RNR3-RT-F	GGGTACAAAATTCTCTGAACAAA	199
RNR3-RT-R	AATGTCACAT TTCTTCTCGTCG	
RNR4-RT-F	ATGGAAGCACATAACCAATTT	455
RNR4-RT-R	GCAATTTCCTTGAATAGAGGGA	
ACT1-RT-F	CACCCTGTTC TTTTGACTGAAGC	520
ACT1-RT-R	TACCGGCAGATTCCAAACCC	
HUG1-RT-F	CCTTAACCCAAAGCAATTCTTCC	190
HUG1-RT-R	TTAGTTGGAAGTATTCTTACCAATGTC	
TSA1-C47S-F	CCATTGGCCT TCACTTTCGTCT(C)TCCAACCGAAATCATTG	
TSA1-C47S-R	CAATGATTTCGGTTGGA(G)AGACGAAAGTGAAGGCCAATGG	
TSA1-C170S-F	AGAACGGTACTGTCTTGCCAT(C)TAACTGGACTCCAGGTGC	
TSA1-C170S-R	GCACCTGGAGTCCAGTTA(G)ATGGCAAGACAGTACCGTTCT	

The nucleotides to be changed are parenthesized.

Plasmid pGal-RNR1 kindly provided by Dr. Stephen Elledge has also been described previously [52].

### Measurement of Mutation Rates, dNTP, and ROS Levels

Rates of spontaneous forward mutations to confer CAN^R^ or 5FC^R^ were measured as described [18],[53]. Spectra of CAN^R^ mutations were determined by DNA sequencing. Ten independent cultures were analyzed in each experiment. Cell extracts were prepared and dNTP levels were measured with Klenow enzyme and [^3^H] labeled dATP or dTTP (PerkinElmer) as described [54]. Standard curves were used to estimate the cellular dNTP levels. Three independent cultures were analyzed in each experiment. Intracellular ROS levels were measured by fluorimetry using DCF (Molecular Probes) as described [4],[18].

### RNA Analysis

Total RNA was extracted by phenol/freeze RNA preparation method as described [55]. For RT-PCR, 3 µg of total RNA was used for cDNA synthesis. Semi-quantitative PCR was performed and optimized to ensure that the amplification was in the linear range. PCR primers were listed in Table 3.

### Western Blotting

Western blot analysis was performed essentially as described [31]. Yeast cells were harvested by centrifugation, followed by trichloroacetic acid extraction with the help of glass beads.
